# The Journey of Data Within a Global Data Sharing Initiative: A Federated 3-Layer Data Analysis Pipeline to Scale Up Multiple Sclerosis Research

**DOI:** 10.2196/48030

**Published:** 2023-11-09

**Authors:** Ashkan Pirmani, Edward De Brouwer, Lotte Geys, Tina Parciak, Yves Moreau, Liesbet M Peeters

**Affiliations:** 1 ESAT STADIUS KU Leuven Leuven Belgium; 2 Biomedical Research Institute Hasselt University Diepenbeek Belgium; 3 Data Science Institute Hasselt University Diepenbeek Belgium; 4 University Multiple Sclerosis Center Hasselt University Diepenbeek Belgium

**Keywords:** data analysis pipeline, federated model sharing, real-world data, evidence-based decision-making, end-to-end pipeline, multiple sclerosis, data analysis, pipeline, data science, federated, neurology, brain, spine, spinal nervous system, neuroscience, data sharing, rare, low prevalence

## Abstract

**Background:**

Investigating low-prevalence diseases such as multiple sclerosis is challenging because of the rather small number of individuals affected by this disease and the scattering of real-world data across numerous data sources. These obstacles impair data integration, standardization, and analysis, which negatively impact the generation of significant meaningful clinical evidence.

**Objective:**

This study aims to present a comprehensive, research question–agnostic, multistakeholder-driven end-to-end data analysis pipeline that accommodates 3 prevalent data-sharing streams: individual data sharing, core data set sharing, and federated model sharing.

**Methods:**

A demand-driven methodology is employed for standardization, followed by 3 streams of data acquisition, a data quality enhancement process, a data integration procedure, and a concluding analysis stage to fulfill real-world data-sharing requirements. This pipeline’s effectiveness was demonstrated through its successful implementation in the COVID-19 and multiple sclerosis global data sharing initiative.

**Results:**

The global data sharing initiative yielded multiple scientific publications and provided extensive worldwide guidance for the community with multiple sclerosis. The pipeline facilitated gathering pertinent data from various sources, accommodating distinct sharing streams and assimilating them into a unified data set for subsequent statistical analysis or secure data examination. This pipeline contributed to the assembly of the largest data set of people with multiple sclerosis infected with COVID-19.

**Conclusions:**

The proposed data analysis pipeline exemplifies the potential of global stakeholder collaboration and underlines the significance of evidence-based decision-making. It serves as a paradigm for how data sharing initiatives can propel advancements in health care, emphasizing its adaptability and capacity to address diverse research inquiries.

## Introduction

Chronic diseases such as multiple sclerosis (MS) [1] present significant obstacles for research, primarily because of their limited prevalence, resulting in smaller study populations [2]. The scarcity of the affected individuals is reinforced when considering the dispersion of real-world data (RWD) across diverse repositories. This scarce RWD, sourced during routine clinical care [3,4], further coupled with heterogeneity in formats, quality standards, and regulatory guidelines, make the comprehensive collection and extraction of meaningful clinical insights even more challenging [5,6].

Despite these challenges, well-managed RWD have the potential to reveal significant patterns concerning diseases, patient experiences, and treatment outcomes [7,8]. For instance, during the early stages of the COVID-19 pandemic, innovative data acquisition strategies overcame data scarcity, unlocking the potential of RWD for meaningful analysis [9-11]. These specific instances underline the broader concern: the RWD landscape is rife with challenges that are often understated.

Current literature tends to oversimplify the intricate processes involved in managing RWD. These include standardization, acquisition, quality enhancement, integration, storage, governance, visualization, and eventual analysis and interpretation. Although these facets are crucial, they are often treated as isolated components rather than integral parts of an interconnected system, with certain areas occasionally overlooked [5].

Recent studies on COVID-19 bring this gap into sharper focus. Khalid and colleagues [12] focused on building analytical models by using observational health data through machine learning but did not fully emphasize the vital aspect of data acquisition in the pipeline management. By contrast, Nishimwe and colleagues [13] concentrated on data integration, gathering data from various hospitals, but did not delve into thorough in-depth data analysis. A study by Junior and colleagues [14] aimed to cover the whole data analytics pipeline but primarily focused on standardizing data from 2 different countries, giving less attention to crucial parts of RWD management, such as data acquisition, preprocessing, quality enhancement, and analysis. This fragmented focus points to the need for a more comprehensive strategy that neither compromises nor overlooks any part of the RWD management process. The absence of a holistic framework, coupled with the growing diversity and volume of RWD sources, intensifies the challenges in health care data sharing and the conversion of RWD into actionable evidence, underscoring the need for standardized management [6].

In light of these challenges, the global data sharing initiative (GDSI) emerges as an exemplary solution that addresses multiple facets of RWD management, specifically in the context of COVID-19 and MS. Prompted by the urgent need to understand COVID-19’s effects on people with MS, GDSI was launched [15]. By integrating data from over 80 countries, GDSI generated globally relevant insights [7,16-19]. This large-scale effort resulted in the formation of the most extensive international cohort of COVID-19 cases among people with MS. In addition to enriching our understanding of the COVID-19 and MS interaction, GDSI showcased the enormous potential of large-scale international collaboration. Furthermore, the initiative set a methodological standard in global health research by introducing a data analysis pipeline with applications beyond MS.

**Figure 1:**
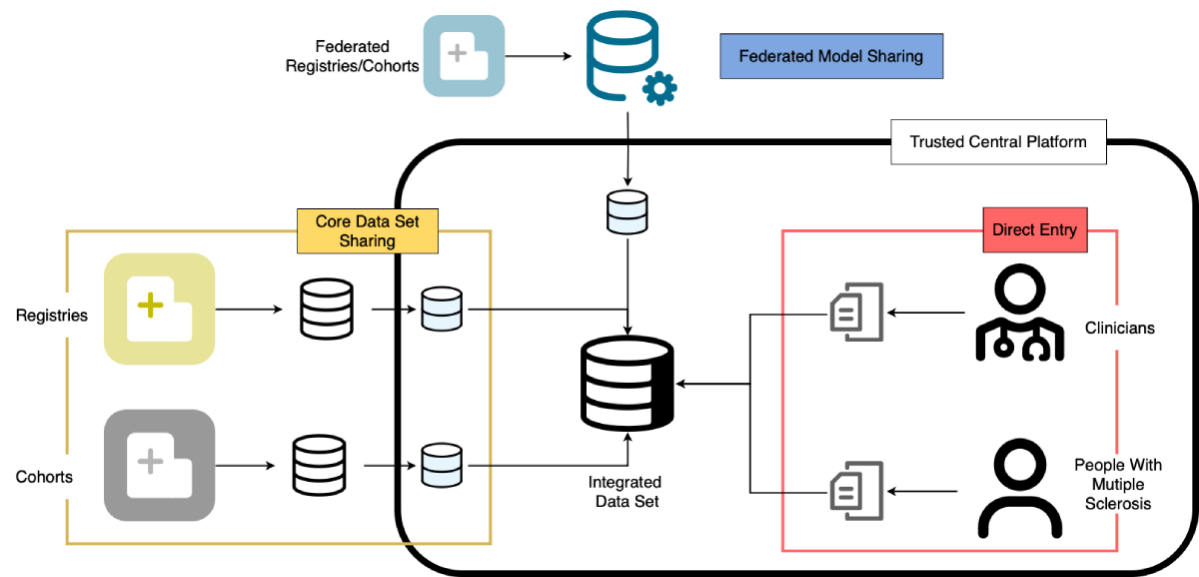
The global data sharing initiative data streams detailing the initiative’s inclusive approach through a hybrid 3-layer data acquisition architecture: (1) direct entry, where individuals upload their data via a web-based form; (2) core data set sharing, where registries upload patient-level data to the central platform under signed data transfer agreements and ethics approvals; and (3) federated model sharing, allowing registries with restrictive policies to participate without directly submitting patient-level data to the central platform.

This paper delves deep into GDSI’s comprehensive RWD analysis pipeline, offering an end-to-end approach that spans from introducing a data dictionary to meticulous data acquisition, and ultimately, to deriving insightful clinical interpretations. One distinguishing aspect of our study lies in the pragmatic execution of this intricate end-to-end analysis pipeline. As depicted in Figure 1, we have implemented a hybrid 3-layer data acquisition architecture—all in strict compliance with the legal and ethical standards that govern data collection and dissemination. Designed for versatility and inclusivity, this architecture aimed to capture every data point possible. Concurrently, an astute approach to data integration was used, whereby these diverse data streams were seamlessly unified. This robust unified data set was then readied for further analysis and exploration.

## Methods

### Overview of GDSI’s Data Analysis Pipeline

**Figure 2:**
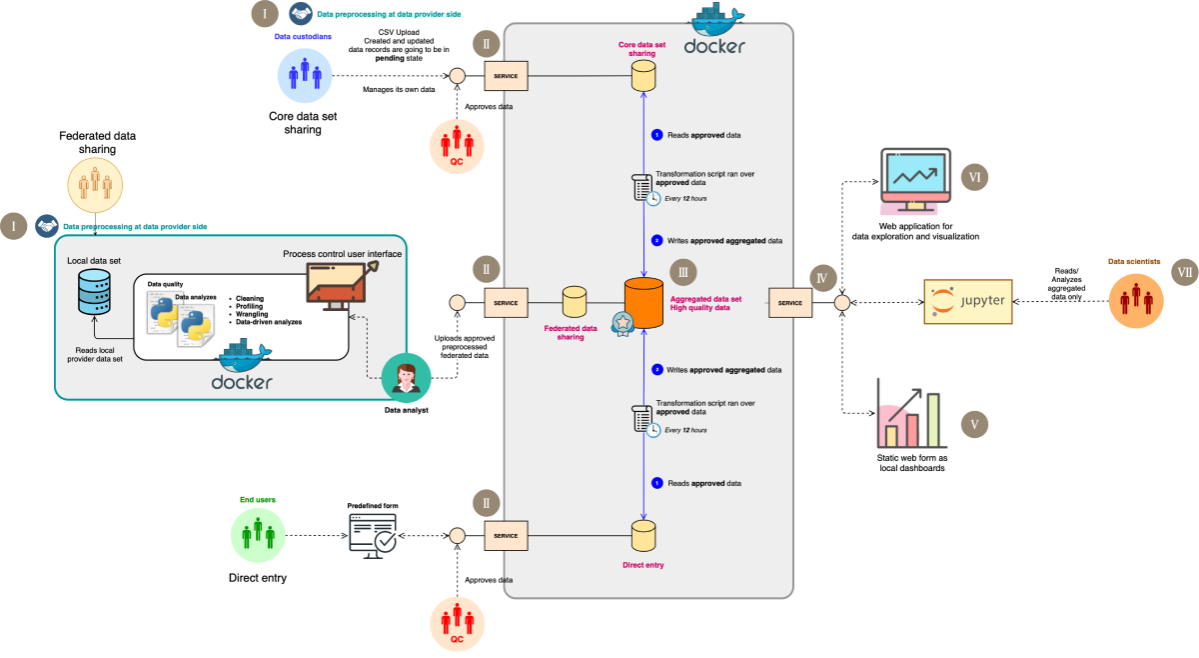
The global data sharing initiative’s end-to-end real-world data analysis pipeline. Step I illustrates the standardization process, which serves as the foundation of this architecture. In this phase, data custodians are requested to map their data to the “COVID-19 in multiple sclerosis core data set” (here referred to as the data dictionary). This process applies only to the core data set and federated model sharing registries, as direct entry is already embedded with a data dictionary via the web form. Step II involves the data acquisition pipeline, featuring distinct levels of data acquisition that depend on the data holder’s willingness and internal policies, all conducted in line with ethical and legal standards. Direct entry, core data set sharing, and federated model sharing constitute the 3 data stream levels. The first 2 levels interact directly with a central platform where the core dataset is shared as static files, in this instance, Comma Separated Values (CSV), whereas federated registries necessitate additional steps before submitting outcomes to the central platform. To incorporate federated registries into the pipeline, predefined queries are dispatched alongside Docker containers to the local side of the registries. The results of these containers are then shipped back to the central platform. In step III, data from different data holders are stored in separate layers to facilitate the next data integration process. Data integration, the subsequent step in the pipeline, entails consolidating data from distinct layers into a comprehensive data set. Step IV emphasizes the utilization of the integrated data set for further data exploration and analysis. Step V highlights the local dashboard, which serves as a quality check, enabling data providers to give feedback on their uploaded data as an additional sanity check. Step VI underscores the online dashboard that has been fed by the integrated data set, utilized by the taskforce during the development of the research questions to ascertain the feasibility of the study and to monitor the data being collected. In step VII, a Jupyter Notebook is provided to the data analysis team, securely connected to the integrated data set, facilitating statistical analysis.

The robustness and scale of GDSI’s endeavor were mirrored by its foundational approach. As depicted in Figure 2, GDSI’s RWD analysis pipeline provided the essential framework for comprehensive data management and analysis. Centralized around a core platform, this pipeline progressed through 5 key stages (1) introducing a specialized data dictionary to standardize the data; (2) data acquisition, which details the methods used to gather the data; (3) an integral step for enhancing data quality; (4) data integration, responsible for aggregating various sources; and (5) the final stage, where the consolidated data are analyzed.

### Ethics Approval

This study received ethics approval from the ethics committee of Hasselt University (approval CME2020/025). For an in-depth discussion concerning ethical authorization, kindly refer to Simpson-Yap et al [17].

### Data Dictionary

A data dictionary serves as a guide that details the attributes of components within an information database, ensuring consistent terminology [20-22]. In the context of GDSI, this tool has proven invaluable for mitigating challenges posed by diverse languages and structures. A task force of domain experts, including epidemiologists, neurologists, and data scientists, reached a consensus on establishing the “COVID-19 in MS Core Data set” data dictionary. This guide served as a keystone for harmonizing data from various sources. To tackle interoperability, data custodians used it as a reference, enabling them to standardize their data sets and streamline the extract-transform-load process. A detailed overview of the variables employed in GDSI is provided by Simpson-Yap and colleagues [7], and a full list is accessible via the GitHub repository [23] and presented in Table S1 of Multimedia Appendix 1.

### Data Acquisition

Recognizing the value of diverse data sources for research outcomes [8,24], GDSI developed a hybrid data acquisition architecture. This framework consists of 3 distinct data sharing streams: direct entry, core data set sharing, and federated model sharing. Each stream is designed to accommodate specific data environments, ensuring a comprehensive and multifaceted collection approach. The primary distinguishing factor among these data sharing streams was the extent of willingness to share clinical records with the central server. In practice, confining data collection to a singular stream would drastically reduce the data volume, making the transition of all contributors to 1 mode unattainable. GDSI’s strength was rooted in its adaptability, effortlessly accommodating these 3 sharing streams and fusing them into a unified data set.

### Data Sharing Streams

#### Direct Entry

This stream prioritized direct engagement with both clinicians and patients. Patients provided their clinical records through structured questionnaires, while clinicians offered their observations after acquiring the necessary permissions. A unique characteristic of this stream was its rapid data entry mechanism. The predefined structure, designed to align with a condensed version of the data dictionary, ensured smooth data integration. Data were submitted via a web-based form on the centralized platform. Importantly, this form upheld patient privacy, excluding specific identifiers and enforcing stringent measures against website cookies and trackers.

#### Core Data Set Sharing

Adhering to the conventional approach for clinical data sharing, data providers contributed a subset of their data set to the central platform. Although this mechanism excelled in handling extensive data, it grappled with challenges related to data collaboration agreements and complex regulatory stipulations. The heterogeneity in the data format further compounded these challenges. However, the architecture of core data set sharing was designed to necessitate the schema of the data dictionary. As a result, data custodians needed to standardize their data format according to the data dictionary to upload their data using this stream. Upon achieving this congruence, custodians used the central platform’s interface—a secure bridge that connected the local infrastructure of the data partners to the main platform—for data upload. For enhanced data security, once uploaded, data extraction is restricted. Additional security measures such as user activity monitoring and stringent access policies were further implemented, ensuring that registry members can only view their specific records, thus preserving data confidentiality. As the pandemic progressed, the registries were periodically invited to contribute their core data sets to the central platform.

#### Federated Model Sharing

Addressing challenges such as strict internal policies that deterred or hindered some registries from sharing clinical records with the central platform, the federated model sharing was introduced. This decentralized solution brings a pivotal shift to regular data sharing streams. Central to this model is the principle of querying data directly at its source, thus eliminating the need to transfer patient-level data. Instead of navigating the nuances of individual patient data, this strategy consolidates multivariable categories into aggregated “buckets.” These buckets are grouped categories where similar data are combined together rather than stored separately. By adopting this approach, potential risks linked to transferring patient-level data are mitigated, and the complexities tied to strict data-sharing agreements are streamlined. A detailed examination of the buckets computation methodology can be viewed in Table S2 of Multimedia Appendix 1 and within the associated GitHub repository [25].

Despite its advantages, the federated model sharing stream introduces its own challenges, especially when remote query executions result in inconsistencies across diverse systems. To compute the buckets, scripts were run locally using Docker [26] containers. Docker containers are self-contained software environments that promote standardization, which helps alleviate the typical technical challenges in such processes. These containers, referred to here as the federated pipelines, were deployed on each registry’s infrastructure and were mounted with data that had already been standardized and aligned with the data dictionary, facilitating seamless execution. After computing these buckets, they were transferred to the central platform. Multiple versions of these Docker containers were used to distribute scripts across the federated model sharing registries.

**Figure 3:**
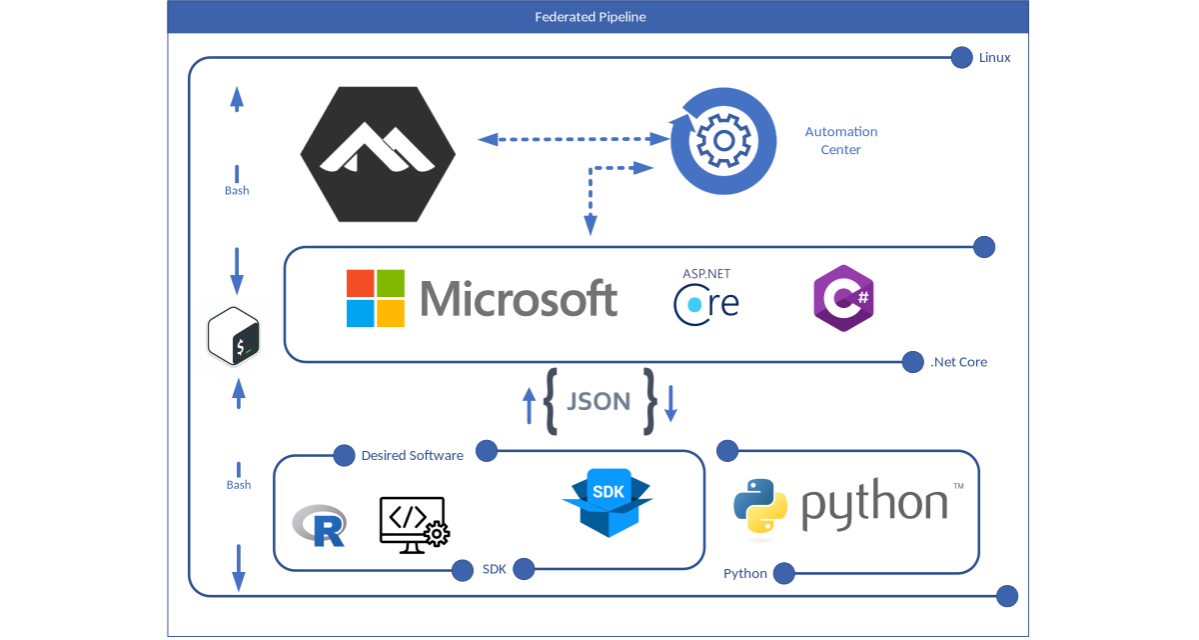
The latest federated pipeline. This is a container composed of 3 primary components. The first component is the base image, which forms the bedrock of the infrastructure. This base image uses Alpine Linux as its underlying operating system, which allows the container to be fine-tuned with other software development kits for further refinements and functionalities [27]. The remaining 2 components, the backend and frontend, are constructed on top of this base image. The backend consists of a suite of Python scripts, which are tasked with data quality assessment, enhancement, cleaning, and analysis. These scripts collaboratively process the incoming mapped data, preparing it for subsequent analysis. By contrast, the frontend was crafted using Microsoft’s ASP.NET Core framework and the C# programming language. Within this pipeline, there is a customizable automation center module. This module can be adapted to meet the specific needs and requests of data partners. It also integrates Crontab, a tool that automates predefined tasks and outlines complex pipelines for execution at various intervals. The automation center module also links the container to the GitHub and Docker Hub version control systems. This connection ensures the use of the most recent scripts and codes published by data analysts. SDK: software development kit.

The architecture of the most recent federated pipeline is presented in Figure 3 [27]. Associated resources, including a demonstrative video walk-through, operational scripts, and the Docker image, can be found in [28-31]. Furthermore, the entire source code has been made publicly accessible on GitHub [32]. This provides a thorough toolkit for those interested in understanding, replicating, or refining the framework of the federated pipeline. A comprehensive analysis of the various iterations of the federated pipeline is presented in Multimedia Appendix 2 [33].

### Data Quality Assessment and Enhancement

The integrity of the acquired data set was upheld through a rigorous data enhancement and quality evaluation process, which was integrated seamlessly into the central platform. In this process, each data variable was scrutinized against a binary criterion: PASS or FAIL. If a specific data point met the pre-established benchmarks of quality and precision, it was accorded a “PASS;” otherwise, it was categorized under “FAIL.” The input format for direct entry eliminated the need for additional quality checks, as validation was directly integrated into the web-based form. For the core data set sharing approach, uploaded data were immediately assessed for quality, and a real-time feedback mechanism alerted contributors to any issues, allowing for immediate corrective action. Conversely, within the federated model sharing approach, quality checks were conducted at the data source prior to aggregation.

The criteria are summarized in Table 1. In the PASS/FAIL column of this table, variables are flagged differently according to the data quality check. PASS is the flag for an accepted variable, FAIL is the flag for a dismissed variable, and EMPTY is the flag for each variable that is missing/null. Note that FAIL does not necessarily mean that the data get excluded; it is just that it is flagged as erroneous—it can also be adapted in some cases for analysis. FAIL means the following action: “Set the FAIL variable to missing, flag the variable, and keep the patient entry (row).” Additionally, specific rules were applied to dates in the data; more specifically, dates cannot be in the future (ie, if any date > date reporting, then the variable is flagged as FAIL) or before a person’s birth date (ie, if any date YEAR < year_reporting - age, then the variable is flagged as FAIL). The COVID-19–related dates also must be later than the MS baseline dates (onset and diagnosis). A comprehensive version of this table is available on GitHub [34]. 

**Table 1:** Data quality assessment and enhancement: pass and fail criteria (some highlighted examples).^a^

Variable	Format	Interdependency	Pass and fail criteria
covid19_date_reporting	yyyy-mm-dd	None	if covid19_date_reporting *<* 2019, then fail, else pass
covid19_has_symptoms	single choice (yes/no)	covid19_sympt_fever covid19_sympt_dry_cough covid19_sympt_fatigue covid19_sympt_pain covid19_sympt_sore_throat covid19_sympt_shortness_breath covid19_sympt_nasal_congestion covid19_sympt_loss_smell_taste covid19_sympt_pneumonia	if covid19_ has_symptoms = null, then check the covid19_sympt_xx for yes if any covid19_symptxx = yes, then covid19 has symptoms = yes (for the analysis data) strict: if covid19_has_symptoms = no AND any covid19_symp_xx = yes, then fail derivation: covid19_has_symptoms is secondary to covid19_ sympt _xx if any of the single symptoms are yes, then empty(!) covid19_has_symptoms will be set to yes and vice versa (all symptoms = no, covid19_has_symptoms is set to no)
covid19_sympt_fever	single choice (yes/no)	covid19_has_symptoms	see covid19_has_symptoms
covid19_sympt_fatigue	single choice (yes/no)	covid19_has_symptoms	see covid19_has_symptoms
covid19_admission_hospital	single choice (yes/no)	None	if covid19_admission_hospital = yes AND covid19_confirmed_case = no, then fail
age_years	Integer	None	if age_years *<* 0 OR age_years *>* 110, then fail, else pass
ms_onset_date^b^	yyyy-mm-dd	ms_diagnosis_date covid19_suspected_onset	if (ms_onset_date *>* ms_diagnosis_date) OR (ms_onset_date *>* covid19_suspected_onset), then fail, else pass
edss_value^c^	Number (0.0, 10.0)	None	if edss_value *<* 0 OR edss_value *>* 10, then fail
type_dmt^d^	Single choice	None	if type_dmt = null AND type_dmt_other = null AND current_dmt = yes, then fail, else pass
has_comorbidities	single choice (yes/no)	None	if has_comorbidities = null AND any_com_xx = yes, then set has_comorbidities = yes (for analysis)

^a^67 more checks have been performed, but these checks are not presented in this table.

^b^MS: multiple sclerosis.

^c^EDSS: Expanded Disability Status Scale.

^d^DMT: disease-modifying therapy.

### Data Integration

The quality-checked data acquired within each stream are stored distinctly, emphasizing the discrete nature of their origins. Consequently, the challenge emerges not just from the acquisition but notably from the critical task of integrating these separate data sets. To derive comprehensive insights, there was a paramount need to coalesce these distinct data sets into a singular unified structure. In the ensuing sections, we outline the process employed to achieve this integration and present a harmonized analytical framework.

Consider *x_i_ =* (*x_i,1_, …., x_i,k_*) to be the list of control and response variables of patient *i* used in the downstream statistical analysis. *N* indicates the total number of patient records and *K* represents the number of variables of interest. For each variable type, we define a list of nonoverlapping ranges ∑_k_ = σ_k_^1^, σ_k_^2^,…, σ_k_^jk^ that partitions the domain of each variable into distinct categories, that is, each variable x_i,k_ can be categorized into a variable y_i,k_ by defining y_i,k_ = j ≡ x_i,k_ ∈σ_k_^j^ with j ∈ {1,…,j_k_}. The data extracted from the federated model sharing registries were then converted into a multivariate contingency table (*S*) of the patient counts for all combinations of all variables—that is, *S* = {(σ_0_, σ_1_,…, σ_K,c_): σ_K_∈∑_k,c_ = ∑_i=1_^N^ *I* [x_i,0_ ∈σ_0_, x_i,1_ ∈σ_1_,…,x_i,k_ ∈σ_k_ ]}, where *I*[.] is the indicator function.

This set is conveniently represented as a table by considering each element of the set as a row and the columns consisting of different variable names and patient counts. This table was subsequently stored on the central platform. The same computation was performed on the direct entry and core data set, as the raw data were available on the central platform, resulting in their specific binned count tables. Finally, all data sources were aggregated by combining their respective binned counts representation *S*. The aggregation was performed by adding the patient counts of each data source for each subgroup of variables. Then, the aggregate set was expanded into a more extended table by repeating each row several times equal to the patient count of that specific row, which resulted in a table *X* ∈R^N×K^, with *K* as the number of variables used in the analysis and *N* as the number of patients.

### Data Analysis

Following the careful integration of a diverse data set, the pivotal challenge lay in deriving actionable insights. The GDSI analysis pipeline was uniquely engineered to remain agnostic to specific clinical research inquiries. Its versatile design permitted any statistical analysis to be executed based on the variables outlined in the data integration table. The efficacy of this approach is exemplified by its application to various research questions, as highlighted in [7,16,17,19]. The analytical approach implemented by Simpson-Yap and colleagues [7] was adopted for the purpose of this paper. A multilevel mixed-effects logistic regression was employed to analyze the aggregated data table. This was performed to assess the association between disease-modifying therapies (DMTs) and several outcomes, including hospitalization, intensive care unit admission, ventilation, and death, while adjusting for variables such as age, sex, MS phenotype, and disability score. The goal of this evaluation was to determine the impact of MS-specific therapies on the severity of COVID-19. This statistical model provided a fine understanding of the complex relationship between these therapies and disease outcomes.

## Results

### Data Acquisition

**Figure 4:**
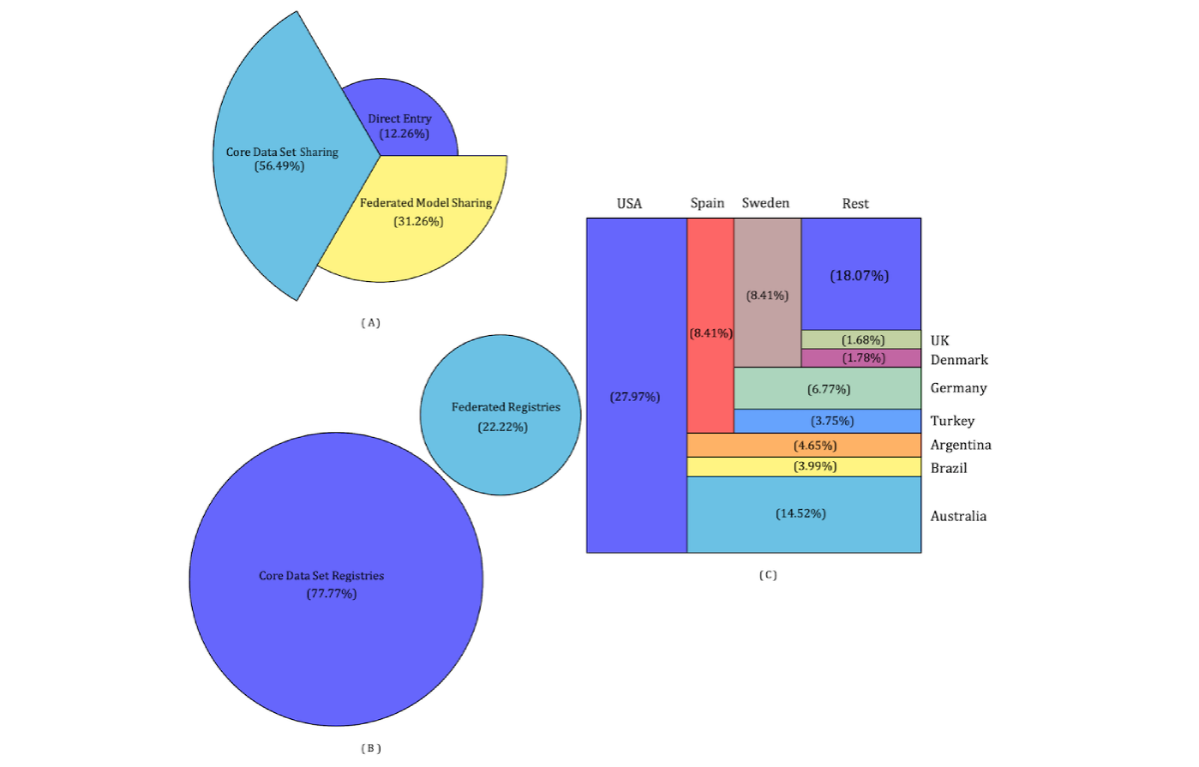
Summary of the data acquired by implementing the 3-layer data acquisition. (A) Federated registries contribute to 31.26% (3527/11,284) of the data, while core data set sharing accounts for 56.49% (6374/11,284). (B) Only 22% (4/18) of the registries participated as federated registries. (C) A summary of the top 10 countries contributing data.

Using the pragmatic 3-layer approach of GDSI, we obtained the largest cohort of people with MS infected with COVID-19. The data were collected from 80 countries, with the top 10 contributing countries being the United States (3157/11,284, 27.97%), Australia (1639/11,284, 14.52%), Spain (949/11,284, 8.41%), Sweden (949/11,284, 8.41%), Germany (765/11,284, 6.77%), Argentina (525/11,284, 4.65%), Brazil (451/11,284, 3.99%), Turkey (424/11,284, 3.75%), Denmark (201/11,284, 1.78%), and the United Kingdom (190/11,284, 1.68%), which accounted for over 80% of the total number of records. Via direct entry, data were collected from 67 countries, with Spain contributing the largest number of records (758/1383, 54.80%), followed by the Netherlands (95/1383, 6.86%), United Kingdom (80/1383, 5.78%), United States (53/1383, 3.83%), Australia (40/1383, 2.89%), and 62 other countries (357/1383, 25.81%), resulting in a total of 1383 records. Data were collected from 18 different registries worldwide. Fourteen of these participated in core data set sharing, contributing to 6374 records. Meanwhile, 4 used the federated model sharing approach, contributing to an additional 3527 records. Table 2 enumerates these data sources. Figure 4 summarizes the number of records acquired at each stream of the data acquisition pipeline. Data acquired through direct entry have been released and are accessible through the associated PhysioNet repository [35].

**Table 2:** Data acquisition summary in the global data sharing initiative (N=11,284).

Method of data sharing	Values, n (%)
Direct entry	1383 (12.26)
Core data set sharing	6374 (56.49)
Federated model sharing	3527 (31.26)

### Data Analysis

Within the data analysis conducted to assess the impact of different DMTs on COVID-19 severity [7], random effects were grouped by data sources. The following variables were used: age, sex, MS phenotype, disability score, DMTs, and COVID-19 severity. Age was categorized in the following ranges: Σ_age_ = (18*-*50 years*,* 50*-*70 years*,* >70 years). Sex was binarized into male and female. MS phenotype was binarized into relapsing-remitting MS and progressive MS. The disability score was dichotomized into Σ_Expanded Disability Status Scale_ = (0*,*6)*,*(6*,*10). DMTs were categorized into Σ_DMT_ = (untreated, alemtuzumab, cladribine, dimethyl fumarate, fingolimod, glatiramer acetate, interferon, natalizumab, ocrelizumab, rituximab, teriflunomide, and other). COVID-19 severity was categorized into Σ_severity_ = (hospitalization, intensive care unit admission, ventilation, death). Compared to patients using all other DMTs, those using rituximab had a higher risk of hospitalization (adjusted odds ratio [aOR] 2.76, 95% CI 1.87-4.07), intensive care unit admission (aOR 4.32, 95% CI 2.27-8.23), and artificial ventilation (aOR 6.15, 95% CI 3.09-12.27). Ocrelizumab showed similar trends for hospitalization (aOR 1.75, 95% CI 1.29-2.38) and intensive care unit admission (aOR 2.55, 95% CI 1.49-4.36) but not ventilation (aOR 1.60, 95% CI 0.82-3.14). Neither rituximab (aOR 1.72, 95% CI 0.58-5.10) nor ocrelizumab (aOR 0.73, 95% CI 0.32-1.70) were significantly associated with the risk of death. A comprehensive report of these findings can be found in Simpson-Yap et al [7].

## Discussion

### Insights From the GDSI Study on MS and COVID-19

The COVID-19 pandemic underscored a pressing need to understand its effect on people with MS. Recognizing the criticality of solid evidence for disease management, a global strategy involving neurologists, patients, and registries was adopted. This collaborative approach paved the way for GDSI’s formation and the development of an end-to-end RWD analysis pipeline. Through this effort, GDSI emerged as the most comprehensive federated international cohort of people with MS impacted by COVID-19, becoming an invaluable resource for informed decision-making. Nevertheless, deriving conclusions from such data initiatives requires careful consideration of the inherent limitations of observational study designs. These studies provide unparalleled real-world insights, but it remains essential to situate the data within the confines of each study’s specific limitations, especially when drawing from post hoc analyses based on existing information [36]. Although GDSI showcased significant advancements, challenges inherent to its structure and execution were encountered. In this section, these challenges are delineated, encompassing aspects from data collection and analysis to concerns of interoperability, data quality, governance, data sharing, and privacy. By exploring these areas, insights are provided to optimize future initiatives and fully harness the potential of RWD in the context of global collaborative learning.

### Challenges and Solutions in Data Interoperability, Quality, and Governance

Interoperability and handling heterogenous data formats presented significant hurdles for GDSI. To counteract these challenges, a study-specific data dictionary was created. However, more advanced standardization methods such as a common data model [37], including Fast Healthcare Interoperability Resources [38] and Observational Medical Outcomes Partnership [39], could further enhance standardization, making it more generalized and disease-agnostic [40,41]. Building on the necessity for standardization, the significance of data quality has been universally recognized in health care, as also highlighted by various studies [42-45]. In tandem with standardization efforts, GDSI integrated an automated data quality assessment framework into its data acquisition process. However, the adoption of a generalized framework such as [46] can serve as a blueprint for enhancing data quality across various health care contexts, providing a more structured format to ensure reliability and precision. As GDSI confronted challenges related to interoperability and data quality, the initiative also had to navigate the complex landscape of regulatory compliance. Implementing a federated governance model, GDSI effectively addressed the existing needs but simultaneously revealed a gap for a data governance model in health care, namely, the absence of implementations specifically tailored for a federated framework [47]. A more universal data governance model such as the one proposed by Peregrina et al [48] could potentially fill this gap, enhancing both organizational efficiency and the quality of analytical models.

### Embracing Federated Model Sharing and Privacy Concerns

Although federated model sharing offers a unique approach to draw insights from patient-level data, it is worth acknowledging that even the impersonal shared statistics inherently encode some information [49]. However, these potential risks are managed under the strict supervision of GDSI, which operates within a rigorously regulated and controlled environment with trusted partners. To further mitigate risks, the data custodians in the federated model sharing underwent a formal assessment of privacy risks after running the script and before sharing the aggregated data with the central platform. This additional layer of scrutiny ensured that any potential privacy concerns were addressed prior to data dissemination. Potential risks and their mitigation strategies were transparently communicated to all data providers via a clear analysis plan, thereby striking a robust balance between efficient data use and strict privacy and security standards. Although GDSI’s federated model sharing has proven successful, it falls short in one crucial area: iterative asynchronous communication. This oversight leads to the introduction of federated learning [50], a methodology wherein a machine learning algorithm extracts knowledge from a variety of locally stored data without the need to transfer raw data enabling deploying sophisticated analysis [51]. Nonetheless, it is vital to recognize the associated risks and challenges. Federated learning or, in general, federated model sharing is not invulnerable to attacks [52,53] or privacy breaches [49].

Considering these risks, it might be necessary to re-evaluate GDSI’s current assumptions of trustworthiness, inquisitiveness, and nonantagonistic behavior among all participants for a wider scope of application. Incorporating privacy-preserving algorithms such as differential privacy [54] and homomorphic encryption [55] can bolster security measures, though potentially affecting analytical performance or necessitating extensive computational resources [56]. Despite these challenges, federated learning has shown promise in a range of studies [57-61]. However, most of these analyses were tailored to specific use cases. There remains a need for a more generalized federated learning pipeline that can be applied broadly, rather than being limited to project-specific applications [62].

Recognizing the inherent risks in the federated approach, GDSI took proactive steps to ensure privacy and build trust within the entire pipeline. In response to concerns regarding privacy and tool reliability, GDSI adopted privacy-by-design principles and utilized certified toolboxes that underwent third-party verification. This approach emphasizes the continual need for assessment and evaluation of privacy safeguards.

### Enhancing Collaboration: User Engagement in the GDSI Pipeline

As GDSI delved deeper into privacy and security measures, it became evident that an improved user experience was pivotal for the pipeline’s success. The intricate nature of the RWD analysis pipeline, coupled with its limited visualization capabilities and an initial oversight in stakeholder inclusion, gave rise to a black box perception. Recognizing the urgent need for better communication and more user-friendly tools, GDSI instituted a dedicated task force. This team took charge from the study’s inception to the formulation of evidence-based guidelines, guaranteeing that every stage aligned with the multifaceted needs of all stakeholders. By doing so, GDSI not only fostered trust and collaboration but also strongly resonated with the project’s overarching principles of engagement and transparency.

The deployment of GDSI’s user-centric interactive web application, complemented by detailed documentation and illustrative visuals, helped demystify the pipeline’s complexity. By offering accessible and user-friendly tools, this approach fostered a more nuanced stakeholder engagement, bridging the divide between intricate operations and approachability. The effectiveness of visualization in health care is supported by various studies [63-66]. Tools such as Jaspersoft [67], Tableau [68], Looker [69], Domo [70], Tibco Spotfire [71], and Power BI [71] offer a business-level data analytics platform, underscoring the significance of converting intricate data sets into comprehensible visuals.

### Pragmatism in GDSI: Balancing Innovation and Adaptation

In the conceptualization and development of GDSI, striking a balance between advanced innovation and practical inclusivity was paramount. This principle was clearly manifested in the design of the data acquisition architecture. Typically, health care frameworks gravitate toward a federated or centralized model. Yet, GDSI embraced a hybrid strategy, seeking to cater to a broad spectrum of users and registries. This novel approach marked a significant departure from the norm, merging technological advancement with operational flexibility.

However, with innovation comes challenges. Although GDSI’s analysis pipeline presents a viable technical solution for collaborative health care learning, it also grappled with broader societal challenges. One salient example was the containerization strategy. Initially promising, it met resistance from certain federated model–sharing registries because of their internal policies. Such challenges underscore the ever-present demand for adaptability amid rapid technological shifts. However, GDSI responded proactively, making the source code available and bolstering it with a comprehensive manual and robust support. Such measures exemplify GDSI’s commitment to reconciling groundbreaking advancements with real-world constraints.

This commitment extended beyond technical challenges. The global reach of GDSI emphasized the importance of resource efficiency, especially in regions with limited internet connectivity. In striving for a global impact, GDSI reiterated its pledge to balance technological progress with practical considerations across diverse geographies. In light of these experiences, one thing becomes clear for the success of initiatives like GDSI: continuous education, proactive stakeholder engagement, and evidence-based demonstrations in controlled environments are not just beneficial, but they are essential.

### GDSI as a Blueprint for Data-Sharing Initiatives in Biomedical Research

GDSI emerged as a pragmatic blueprint for interdisciplinary biomedical research. The meticulous planning and systematic execution of the initiative showcased how strategic processes can serve as foundational guides for upcoming biomedical consortia. The open-source resources GDSI provides [23,25,29,30,32,34,35,72-74] can be directly leveraged and adjusted after thorough assessment and evaluation. These resources bifurcate into 2 main categories: disease-agnostic and disease-specific components.

Within the context of disease-agnostic components, the architecture of GDSI’s end-to-end data analysis pipeline stands out, highlighting its modularity and adaptability. This pipeline’s design facilitates significant customization, catering to various data acquisition streams. The hybrid nature of the data acquisition module allows initiatives to choose one or a combination of data collection methods based on their distinct needs and policies. Additionally, GDSI’s data integration framework plays a crucial role in amalgamating these diverse data sources into a unified and comprehensive data set. Together, these components offer a versatile foundation that other biomedical initiatives can adapt and leverage according to their specific requirements.

Turning to disease-specific components, aspects like the data dictionary and data quality assessments were designed primarily for the research question centered around MS and COVID-19. Even though these components are specialized, they act as guiding principles for other research ventures. The data dictionary, augmented by its metadata, provides a robust foundation for the next phases of the pipeline. It offers a detailed account of acquisition variables and sets clear data quality criteria. A significant point to note is that the data dictionary aids in determining the rules for data quality assessments, presenting a methodical approach to data validation. This thorough approach emphasizes the importance of precise planning and specificity when delving into disease-focused research questions, setting an example for other initiatives to follow.

To conclude, the flexibility and adaptability inherent in GDSI’s comprehensive data analysis pipeline coupled with its disease-specific components meld to present a versatile tool for crafting sturdy data architectures across a spectrum of biomedical research landscapes. Those seeking a deeper understanding and guidance on harnessing and replicating GDSI’s capabilities can refer to Multimedia Appendix 3, which offers a detailed roadmap based on GDSI’s experiences and insights, a flowchart tracing the initiative from its inception to its research question resolutions, and guidance on replicating GDSI’s federated model sharing infrastructure. A graphical abstract delineating the high-level architecture of this study is presented in Multimedia Appendix 4, which provides additional insights regarding the architectural framework.

### Conclusion

GDSI had substantial impact that extended beyond its initial focus on COVID-19 and MS. It contributed to numerous scientific publications and played a pivotal role in shaping global guidelines for the community with MS [7,9,16-19]. This underscores the vast potential of data-driven collaborative efforts to yield improved health care outcomes. A cornerstone of GDSI’s success was its RWD analysis pipeline. Crafted to navigate technical, epidemiological, and sociological challenges, this pipeline facilitated the seamless integration of varied data streams into a single data set. This cohesive strategy enabled large-scale collaborative research and offered the flexibility to accommodate the diverse policies, regulations, and needs of various data providers. Serving as a practical blueprint, GDSI addressed not only current health care challenges but also laid the groundwork for future initiatives. Its hybrid approach to data acquisition and analysis provided a scalable framework applicable to other health care sectors. In doing so, GDSI stands as a compelling example of how data sharing and collaborative learning can meaningfully advance health care research, going beyond the specific challenges of MS and COVID-19.

## Appendix

Multimedia Appendix 1 Analysis of the data dictionary employed within the global data sharing initiative.

Multimedia Appendix 2 The 3 iterative pipelines developed: federated pipeline COV1.0, COV2.0, and COV2.1.

Multimedia Appendix 3 Roadmap of the global data sharing initiative.

Multimedia Appendix 4 Graphical abstract.

## Abbreviations

aOR: adjusted odds ratio
DMT: disease-modifying therapy
GDSI: global data sharing initiative
MS: multiple sclerosis
RWD: real-world data
