# Supplementary material for: The Journey of Data Within a Global Data Sharing Initiative: A Federated 3-Layer Data Analysis Pipeline to Scale Up Multiple Sclerosis Research
Source: JMIR Med Inform. 2023 Nov 9;11:e48030. doi: 10.2196/48030 (PMC10667980; doi:10.2196/48030)
Supplement: Multimedia Appendix 1 [file medinform_v11i1e48030_app1.docx]

## Multimedia Appendix 1

In this part, we offer a detailed analysis of the data dictionary employed within the GDSI. Additionally, our discussion broadens to encompass the architectural schema of the federated model sharing in relation to the sharing of variables, as well as the computational methods linked to this process.

By elucidating both the data dictionary and the federated model sharing schema, our objective is to impart a thorough understanding of the systematic organization, interrelationships, and manipulation techniques of data within the GDSI framework.

A summary of the dictionary is also shown in Table S1. The variables of the data dictionary are clustered into six groups: (1) COVID-19 incidence, (2) COVID-19 severity, (3) demographics, (4) MS history and severity, (5) DMT information, and (6) comorbidities.

Table S1: Summary of the data dictionary. The collected variables are clustered in six small subsets, and five different features are collected for each variable. Variable Name is the first feature, and it explains which data was collected. Variable ID is how information is stored in the database. Data Type shows the data format, and Value states which values this feature can take. The last feature represents whether that variable was collected by the data partner or not.

| Variable Name | Variable ID | Data Type | Value | Variable Collected |
| --- | --- | --- | --- | --- |
| COVID-19 Incidence |  |  |  |  |
| Date of Visit/Reporting | covid19_date_reporting | YYYY-MM-DD | Date | Yes/No |
| COVID-19 Symptoms | covid19_has_symptoms | Single Choice | Binary | Yes/No |
| Duration of self-isolation (in days) | covid19_self isolation_duration | Number | Integer | Yes/No |
| country COVID-19 symptoms occurred | covid19 country | Text | Text | Yes/No |
| *+17 more fields* |  |  |  |  |
| COVID-19 Severity |  |  |  |  |
| Admission in Hospital of COVID-19 | covid19_admission_hospital | Single Choice | Binary | Yes/No |
| Admission date | covid19_admission_hospital_date | YYYY-MM-DD | Date | Yes/No |
| Ventilation needed during hospital stay? | covid19_ventilation | Single Choice | Binary | Yes/No |
| Did the patient receive ECMO? | covid19_ecmo | Single Choice | Binary | Yes/No |
| *+10 more fields* |  |  |  |  |
| Demographics |  |  |  |  |
| Age (years) | Age_years | Number | Integer | Yes/No |
| Sex | Sex | Single Choice | Options | Yes/No |
| Current Smoker | Current_smoker | Single Choice | Binary | Yes/No |
| Weight (in kg) | Weight | Number | Integer | Yes/No |
| *+4 more field* |  |  |  |  |
| MS history and severity |  |  |  |  |
| MS Type | Ms_type | Single Choice | Options | Yes/No |
| MS onset | Ms_onset_date | YYYY-MM-DD | Date | Yes/No |
| EDSS value | Edss_value | Number | Float [0.0,10.0] | Yes/No |
| Date of evaluation | Edss_date_diagnosis | YYYY-MM-DD | Date | Yes/No |
| *+7 more fields* |  |  |  |  |
| DMT Information |  |  |  |  |
| DMT current usage | Current_dmt | Single Choice | Options | Yes/No |
| Type of last/current DMT | Type_dmt | Single Choice | Options | Yes/No |
| Glucocorticoid during the past months | Dmt_glucocorticoid | Single Choice | Binary | Yes/No |
| DMT Start Date | Dmt_start_date | YYYY-MM-DD | Date | Yes/No |
| *+8 more fields* |  |  |  |  |
| Comorbidities |  |  |  |  |
| Comorbidities | Has_comorbidities | Single Choice | Binary | Yes/No |
| Cardiovascular disease | Com_cardiovascular_disease | Single Choice | Binary | Yes/No |
| Hypertension | Com_hypertension | Single Choice | Binary | Yes/No |
| Diabetes | Com_diabetes | Single Choice | Binary | Yes/No |
| *+7 more fields* |  |  |  |  |

Table S2 illustrates how buckets were calculated for the federated registries.

Table S2: Data computed for the federated model sharing registries

| Title | Variable ID | How Calculated |
| --- | --- | --- |
| Diagnosis of COVID-19 | covid19_diagnosis | Covid19_diagnosis= “confirmed”;  if covid19_confirmed_case= “yes” Covid19_diagnosis= “suspected”;  if covid19_suspected_case= “yes” |
| Age categories | age_in_cat | age_in_cat = 0 if 0 *<* age_years *<*18  age_in_cat = 1; if 18≤ age_years ≤50  age_in_cat = 2; if 50*<* age_years ≤70  age_in_cat = 3; if 70*<* age_years |
| Type of MS | ms_type2 | ms_type2 = “relapsing_remitting”  if ms_type= “RRMS”  ms type2= “other” if – ms_type= “CIS”  - or ms_type = “not_sure”  - or ms_type = “”  ms_type2= “progressive_MS” if  ms_type= “SPMS”  or ms_type = “PPMS” |
| EDSS Categories | edss_in_cat2 | edss_in_cat2= “zero” if 0≤ edss ≤6  edss_in_cat2= “one” if 6*<* edss |
| DMT Type | dmt_type_overall | dmt_type overall= “No information on DMT use”  if current_dmt= missing or type_dmt = missing  dmt_type_overall= “currently not using any DMT”  if current_dmt= “no” OR “no, but was in the past”  dmt_type_overal= “currently on interferon”  if current_dmt = “yes” AND type_dmt = “interferons”  dmt_type_overall= “currently on glatiramer”  if current_dmt = “yes” AND type_dmt = “glatiramer”  dmt_type_overall= “currently on natalizumab”  if current_dmt = “yes” AND type_dmt = “natalizumab”  dmt_type_overall= “currently on fingolimod”  if current_dmt = “yes” AND type_dmt = “fingolimod”  dmt_type_overall = “currently on dimethyl fumarate”  if current_dmt = “yes” AND type_dmt = “dimethyl fumarate”  dmt_type_overall = “currently on teriflunomide”  if current_dmt = “yes” AND type_dmt = “teriflunomide”  dmt_type_overall = “currently on alemtuzumab”  if current_dmt = “yes” AND type_dmt = “alemtuzumab”  dmt_type_overall= “currently on cladribine”  if current_dmt = “yes” AND type_dmt = “cladribine”  dmt_type_overall = “currently on siponimod”  if current_dmt = “yes” AND type_dmt = “siponimod”  dmt_type_overall = “currently on rituximab”  if current_dmt = “yes” AND type_dmt = “rituximab”  dmt_type_overall = “currently on ocrelizumab”  if current_dmt = “yes” AND type_dmt = “ocrelizumab”  dmt_type_overall = “currently on another drug not listed”  if current_dmt “yes” AND type_dmt other is not missing |
| +15 more variables |  |  |
