# Supplementary material for: The Journey of Data Within a Global Data Sharing Initiative: A Federated 3-Layer Data Analysis Pipeline to Scale Up Multiple Sclerosis Research
Source: JMIR Med Inform. 2023 Nov 9;11:e48030. doi: 10.2196/48030 (PMC10667980; doi:10.2196/48030)
Supplement: Multimedia Appendix 2 [file medinform_v11i1e48030_app2.docx]

## Multimedia Appendix 2

During the progression of the GDSI project, three iterative pipelines were developed—Federated Pipeline COV1.0, COV2.0, and COV2.1—each building upon the features and capabilities of the previous version. These pipelines were designed to cater to the needs of federated model sharing registries, continually improving over time. In the following sections, we will first highlight the key aspects of these pipelines and then provide a detailed overview of the infrastructure, functionalities, and modules associated with them, showcasing their evolution and enhancement over time.

Key infrastructural highlights encompass the following:

1. **Federated Pipeline COV 1.0**: Containerization with employing the Jupyter Data Science Notebook's[75] official Docker image, facilitating a consistent environment for data custodians and ensuring seamless deployment across diverse computing infrastructures.
2. **Federated Pipeline COV 2.0**: Implementation of a language-agnostic base image with Ubuntu Linux as the foundation, a user-friendly web application utilizing the ASP.NET Core framework [76], and the incorporation of an Automation Center module with a job scheduler (Crontab [77]) and seamless connection to version control systems (GitHub and DockerHub).
3. **Federated Pipeline COV 2.1**: Emphasis on privacy and security enhancements through the adoption of Alpine Linux [78], as the container's base image, the execution of multiple hardening processes, and a significant reduction in the container's compressed image size.

The initial iteration, Federated Pipeline COV 1.0, aimed to tackle the complexities associated with computing infrastructure compatibility in federated model sharing. By leveraging containerization and utilizing the official Docker image of the Jupyter Data Science Notebook [75], the pipeline created a consistent, encapsulated environment for data custodians. This approach facilitated compatibility and streamlined deployment across various computing infrastructures, contributing to the pipeline's scalability and adaptability. Upon completion of the analysis, data custodians compiled the results into a compressed zip file and shared them with the data analyst team, which was then manually uploaded to the platform. This process ensured the privacy and security of patient-level data at the local data custodian level while transmitting only aggregated outcomes. Consequently, the first version of the federated pipeline was established, as illustrated in Figure S1.


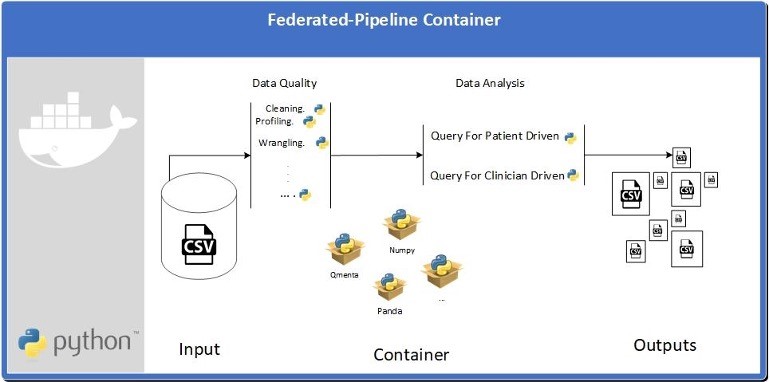


Figure S1: The federated pipeline COV 1.0 architecture.
This infrastructure encompasses a series of scripts written in Python. These scripts are divided into two parts: (1) data quality scripts that are responsible for enhancing and cleaning the data and (2) a predefined query for data analysis.

To enhance the user experience, Federated Pipeline COV 2.0 was developed. The improved architecture featured Ubuntu Linux as the underlying operating system, which enabled the creation of a web service application using the lightweight, cross-platform ASP.NET Core framework. This development offered data custodians a more transparent and user-friendly interface by incorporating different interactive infographics and plots as a result of executing the analyses, reducing the black-box approach associated with federated registries. The backend consisted of a suite of Python scripts for data quality assessment, enhancement, cleaning, and analysis. Key improvements in COV 2.0 included the customizable *Automation Center* module, which could be tailored to the needs and requests of data partners, and the integration of Crontab to automate predefined tasks and define complex pipelines for the execution at different intervals. Additionally, the automation center module connected the container to the version control systems GitHub and DockerHub, ensuring the use of the latest available scripts and codes published by data analysts. Another benefit of the refined architecture was the language-agnostic infrastructure, which allowed data analyst teams to utilize their preferred programming language, eliminating the need for translation. Figure S2 illustrates the updated architecture of the pipeline.


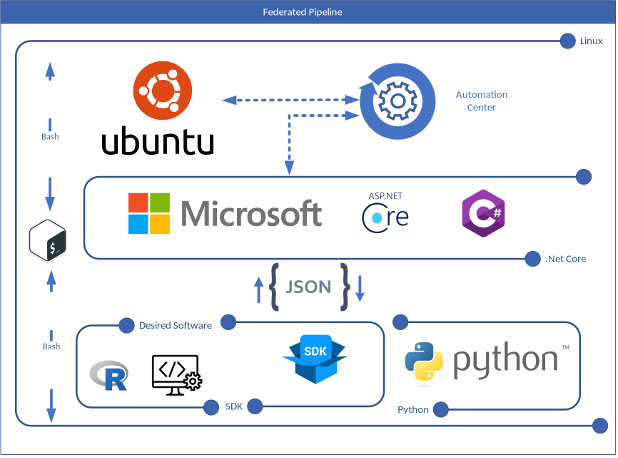


Figure S2: The Federated Pipeline COV 2.0 architecture. This container is comprised of three main components. The first component is the base image, which serves as the foundation of this infrastructure. This base image uses Ubuntu Linux as the underlying operating system. The second and third components, the backend and frontend, are built upon the base image. The backend is composed of a series of scripts written in Python, which are responsible for data quality assessment, enhancement, cleaning, and analysis. These scripts work in tandem to process the incoming mapped data and prepare it for further analysis. The frontend, by contrast, was developed using Microsoft’s ASP.NET Core framework and the C# programming language.

The final iteration, Federated Pipeline COV 2.1, placed significant emphasis on enhancing privacy and security measures because of the sensitive nature of patient-level data interaction. To achieve this, the base image of the container transitioned to Alpine Linux which is renowned for its robust security and lightweight size. Several hardening processes were implemented, including the removal of unnecessary accounts, dangerous commands, and utilities, as well as the restriction of system file and directory permissions. A third-party tool, Snyk [76], was used to evaluate vulnerabilities and privacy risks within the container. The findings of this assessment are summarized in Table S3 .

The transition from Ubuntu to Alpine Linux, combined with the use of minimal resources, specifically in the frontend, resulted in a drastically reduced container image size from 1.31 GB in the initial version to a mere 120 MB in the latest version, representing a decrease exceeding 90%. This led to improved resource efficiency.

The design of the federated model sharing infrastructure was governed by four main principles: privacy, security, efficiency, and user-friendliness. These guiding principles ensured a balanced approach towards efficient data utilization while maintaining stringent standards of privacy, security and facilitating a user-friendly interface.

Table S3: Snyk vulnerability assessment report. This report was exported for the federated infrastructures. The counts are extracted from Snyk. Severity levels are used to evaluate the potential risk posed to an application. A high severity level indicates that an attacker may be able to gain unauthorized access to sensitive data within the application. A medium severity level suggests that under certain circumstances, an attacker could access sensitive data within the application. A low severity level means that the application may expose some data, which could be used alongside other vulnerabilities to launch an attack on the application. These numbers correspond to the privacy and security notice scanned within this platform.

|  | Federated Pipeline  COV 1.0 | Federated Pipeline  COV 2.0 | Federated Pipeline  COV 2.1 |
| --- | --- | --- | --- |
| High | 113 | 19 | 0 |
| Medium | 51 | 12 | 0 |
| Low | 228 | 131 | 0 |
| Total | 392 | 162 | 0 |

Table S4 provides a high-level summary of the infrastructure used in the federated model sharing registries, along with a comparison of the different components within the infrastructure.

Table S4: Overview of the federated infrastructures deployed in the GDSI

|  | COV 1.0 | COV 2.0 | COV 2.1 | Remark |
| --- | --- | --- | --- | --- |
| Containerization | Docker | Docker | Docker |  |
| Base Image | Jupyter | Ubuntu | Alpine |  |
| Interactive Interface | Yes | Yes | Yes | ASP .NET Core web app |
| Interface Technology | Notebook | ASP .NET Core | ASP .NET Core |  |
| Open-source | Yes | Yes | Yes | Under different licenses |
| Language agnostic | No | Yes | Yes | Executes multiple languages |
| Debugging Panel | Yes | Yes | Yes | Notebook interface and Linux shell |
| Customizable | No | Yes | Yes | Based on user requests |
| Version Control | No | No | Yes | Handled by Docker Hub |
| GitHub Repository | No | Yes | Yes | Full development process committed in the repository |
| Job Scheduler | No | Yes | Yes | Scheduling using Cron module |
| Resource Efficiency | 1.31 GB | 334.91 MB | 120.46 MB | 91% improvement |
