# Supplementary material for: The Journey of Data Within a Global Data Sharing Initiative: A Federated 3-Layer Data Analysis Pipeline to Scale Up Multiple Sclerosis Research
Source: JMIR Med Inform. 2023 Nov 9;11:e48030. doi: 10.2196/48030 (PMC10667980; doi:10.2196/48030)
Supplement: Multimedia Appendix 3 [file medinform_v11i1e48030_app3.docx]

## Multimedia Appendix 3

This section aims to offer a comprehensive guide on leveraging the GDSI framework for other research questions, emphasizing the advantages for emerging data-sharing consortia. To tailor GDSI to other disease domains, it is recommended to follow this roadmap:

1. **Establish a Taskforce:** Convene a multidisciplinary team of experts from fields such as data science, clinical research, and epidemiology to provide oversight and guidance throughout the adaptation process.
2. **Analysis Plan Formation:** Develop a robust analysis plan that outlines the study design, objectives, scope, comprehensive research questions, and expected deliverables. The analysis plan should be approved by the taskforce to ensure alignment with the initiative's goals.
3. **Data Dictionary or Harmonization Method:** Create or adapt a comprehensive data dictionary under the supervision of the taskforce. This dictionary should cover all required variables and include metadata like acceptable ranges and input types. Alternatively, integrate a sophisticated data harmonization method if a data dictionary is not feasible.
4. **Customization of Data Acquisition Modules:** Leverage the modularity of GDSI's data acquisition architecture to select, drop, or tailor specific data streams based on the project's scale, objectives, and requirements.
5. **Quality Control Adaptation:** Implement data quality assessments informed by the newly crafted or adapted data dictionary. The metadata in the data dictionary should serve as a cornerstone for defining quality control metrics. Logical controls should also be tailored to align with the objectives outlined in the analysis plan.
6. **Data Integration:** In case of using federated model sharing, use the schema of the particular data stream as the reference point for integration. If federated model sharing is not used, establish core dataset sharing, to serve as the base schema for integrating additional data streams.
7. **Data Visualization and Dashboarding:** Design real-time dashboards and reporting tools for ongoing monitoring. These tools should facilitate sanity checks among stakeholders and the taskforce to ensure the integrity of the collected data and the progress of the pipeline.
8. **Data Analysis**: Conduct data analytics as per the guidelines defined in the analysis plan. Note that the integrated dataset should allow the flexibility for unplanned, exploratory analyses.
9. **Pilot Testing**: Validate the system's performance and adaptability through pilot testing, which can be performed using available benchmark datasets or synthetic data.
10. **Review and Iteration**: Based on feedback and outcomes from the pilot testing, make any necessary refinements to prepare the pipeline for broader-scale deployment.

Figure S3 elucidates the roadmap of GDSI by outlining the critical steps from the initiative's inception to the realization of its intended outcomes.


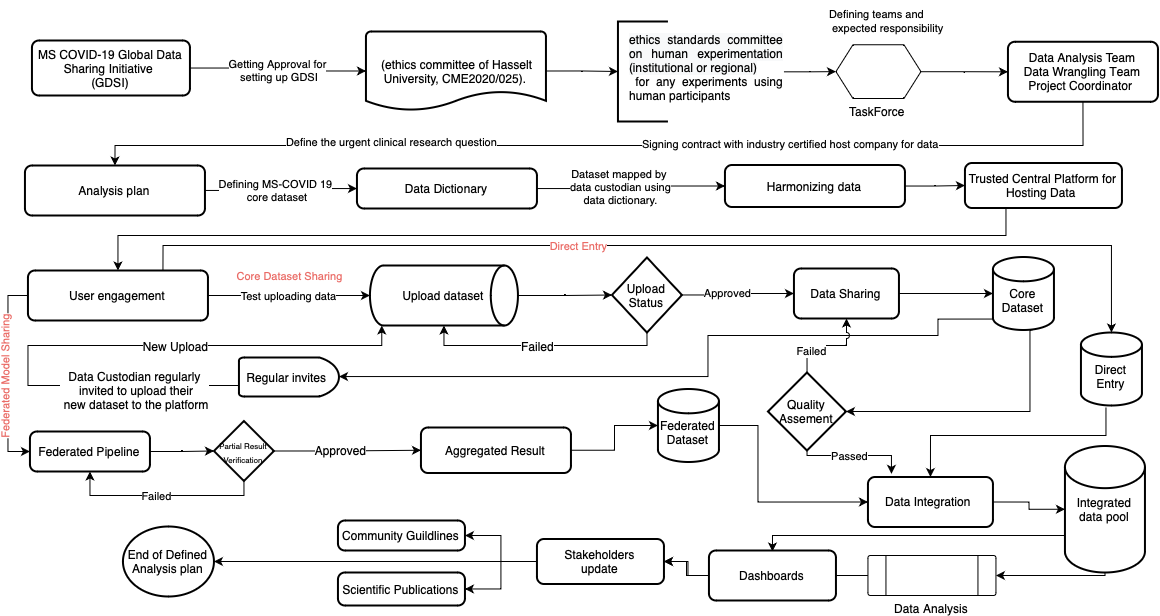


**Figure S3:** GDSI Flowchart: A Comprehensive Roadmap Detailing the Key Steps from Initiative Inception to Final Outcomes, Serving as a Blueprint for Adapting GDSI Principles to Other Biomedical Data Sharing Consortia

In the absence of GDSI's central platform, a federated model sharing infrastructure alongside a direct entry dataset [35] can co-create a smaller scale version of GDSI. Moreover, a tunable mock data generator [74] has been provided to create datasets of various sizes and characteristics, acting as a synthetic registry. This mock dataset can be integrated into the federated model sharing infrastructure, and the aggregated results can be used to produce a unified dataset for various analyses, as demonstrated in Figure S4.


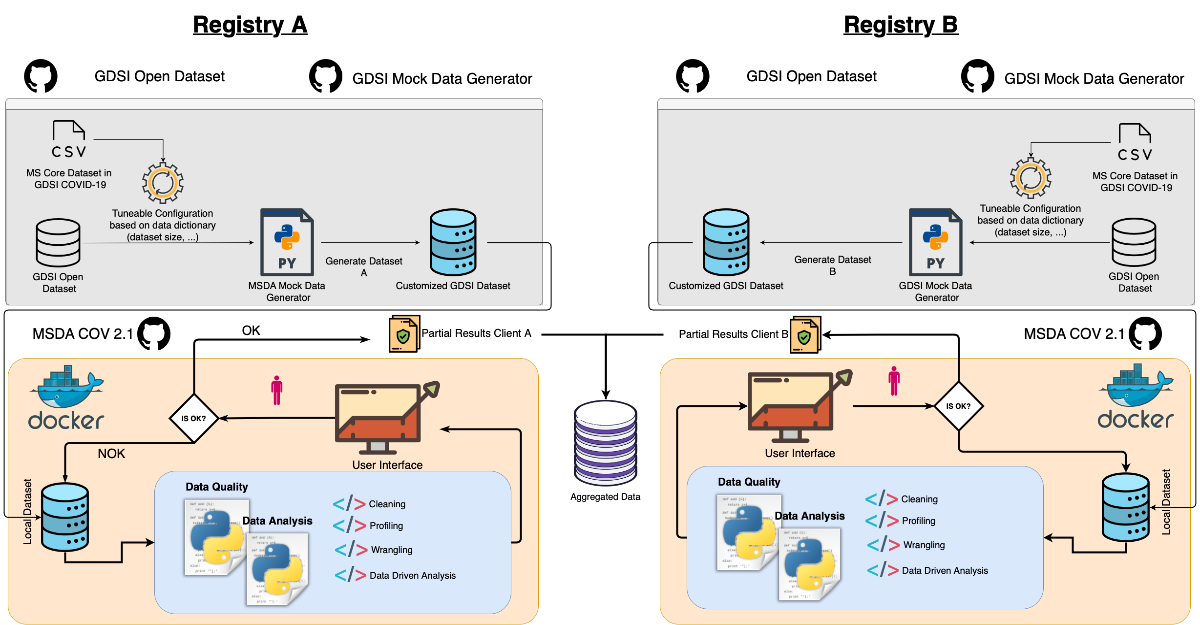


**Figure S4:** A Guide to Replicating Federated Model Sharing Using GDSI Principles
