# Supplementary figures and images for: The Journey of Data Within a Global Data Sharing Initiative: A Federated 3-Layer Data Analysis Pipeline to Scale Up Multiple Sclerosis Research
Source: JMIR Med Inform. 2023 Nov 9;11:e48030. doi: 10.2196/48030 (PMC10667980; doi:10.2196/48030)

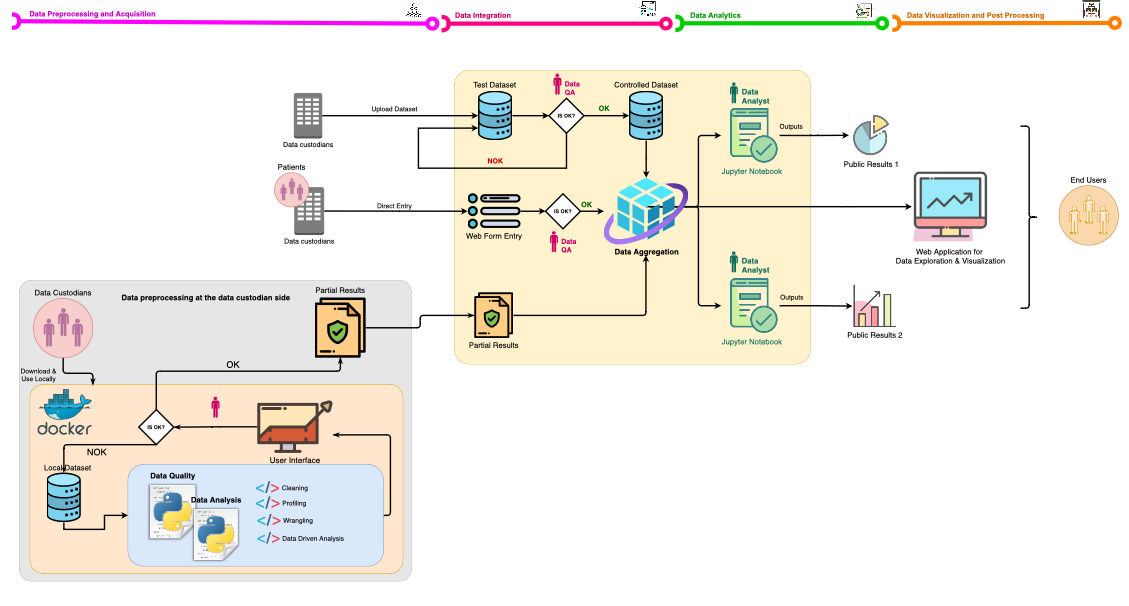

Supplement: Multimedia Appendix 4 [file medinform_v11i1e48030_app4.png]
